# Highly proliferative anal neuroendocrine carcinoma: molecular and clinical features of a rare, recurrent case in complete remission

**DOI:** 10.1186/s12876-020-01433-6

**Published:** 2020-08-27

**Authors:** Carl Christofer Juhlin, Henrik Falhammar, Magnus Kjellman, Jan Åhlén, Staffan Welin, Jan Calissendorff

**Affiliations:** 1 Department of Oncology-Pathology, BioClinicum J6:20, Karolinska Institutet, Stockholm, Solna, Sweden; 2 Department of Pathology and Cytology, Karolinska University Hospital, Stockholm, Sweden; 3 Department of Endocrinology, Metabolism and Diabetes, Karolinska University Hospital, Stockholm, Sweden; 4 Department of Molecular Medicine and Surgery, Karolinska Institutet, Stockholm, Sweden; 5 Department of Breast and Endocrine Surgery, Karolinska University Hospital, Stockholm, Sweden; 6 Institution of Medical Sciences, Uppsala Akademiska Hospital, Uppsala, Sweden; 7 Department of Endocrine Oncology, Uppsala Akademiska Hospital, Uppsala, Sweden

**Keywords:** Anal neuroendocrine carcinoma, Remission, HPV, PIK3CA, Mutation, Case report

## Abstract

**Background:**

Poorly differentiated anal neuroendocrine carcinomas (ANECs) are rare lesions with poor prognosis, and the molecular etiology is only partially understood.

**Case presentation:**

At our institution, we have treated and followed a patient with such a rare ANEC. He had primarily surgery followed by three rounds of repeated surgery for loco-regional recurrences. He also received three different combinations of chemotherapy and external beam radiation. At last follow-up 13 years since the primary diagnosis, the patient had been in complete remission for nine years.

The patient’s medical files were re-examined, including laboratory, radiology and clinical examinations. Histopathology was re-assessed, and expanded immunohistochemistry was performed from tissue specimens from the four surgical procedures. In addition, the molecular genetic status was evaluated through next-generation sequencing.

The initial tumor was consistent with a 59 mm small cell neuroendocrine cancer with a Ki-67 index of 80%. Regional lymph node metastases were evident, and immunohistochemistry supported a neuroendocrine origin. A PCR screening detected human papilloma virus type 45 DNA (high-risk subtype), and focused next-generation sequencing found a missense mutation in the *Phosphatidylinositol-4,5-Bisphosphate 3-Kinase Catalytic Subunit Alpha* (*PIK3CA*) gene. In tissues representing subsequent recurrences, the Chromogranin A expression was lost, and the Ki-67 index increased to 90%.

**Conclusions:**

For the first time, we report the detection of HPV45 in a case of ANEC. To our belief, *PIK3CA* mutations have also not been previously demonstrated in this tumor entity. In highly malignant ANECs, cure can in rare cases be achieved. Although speculative, expression of HPV45 and/or the *PIK3CA* mutation may have contributed to the favorable outcome.

## Background

Carcinomas of the anal tract are mainly squamous cell carcinoma (SCC) or adenocarcinomas, with anal neuroendocrine carcinomas (ANECs) representing only 1% of anal malignancies [1]. Small, localized and well-differentiated neuroendocrine tumors are much more common, are usually < 10 mm in size and have rarely invaded or metastasized at diagnosis [2]. Neoplasms with neuroendocrine features are graded by a Ki-67 proliferation index, ranging from G1 (Ki-67 index < 3%), G2 (3–20%) and G3 (> 20%). G3 tumors are further subdivided into well-differentiated NET G3 and NECs [3]. The pathogenesis is unclear, but ANECs can occur synchronously with squamous cell cancer [4] or adenocarcinoma [5], but does not seem to develop as part of a dedifferentiation from well-differentiated neuroendocrine tumors [6].

Treatment with surgery and/or chemotherapy is normally preferred, initially often platinum-etoposide or based on 5-fluoruracil [7], but the outcome is generally poor, and the five-year survival rate has been estimated to 3–27% when distant metastases are present [8, 9]. Due to the rarity of ANECs, prospective studies are lacking and no standard treatment regimen has been developed. Moreover, guidelines as to if surgery should be performed unless the tumor is localized, or if chemotherapy should be the therapy of choice, are also lacking.

At our institution, we have now treated and followed a patient diagnosed with ANEC during 13 years. This male patient described herein has had surgery four times, and has been treated with chemotherapy and external radiation, and is now recurrence-free. Our aim is to describe the clinical and histopathological features of this highly malignant tumor presenting with a successful long-term outcome. The patient’s medical files were re-examined, including laboratory, radiology and clinical examinations. Histopathology was re-assessed, and expanded immunohistochemistry was performed from tissue specimens from the four surgical procedures.

All immunohistochemical stainings were carried out in our pathology laboratory using the Ventana Benchmark Ultra system (Ventana Medical Systems, Tucson, AZ, USA). All stainings were analyzed by an endocrine pathologist (CCJ).

DNA extraction from formalin-fixated paraffin-embedded (FFPE) tissues was performed using the Maxwell® DNA FFPE Tissue kit from Promega, and the Oncomine Solid Tumor Panel (Ion Torrent S5, Hi-Q Chef; Thermo Scientific) was used to screen for > 1800 cancer related mutations within the following genes: *EGFR, KRAS, NRAS, PIK3CA, BRAF. ALK, ERBB2, ERBB4, FGFR1, FGFR2, FGFR3, MET, DDR2, AKT1, PTEN, MAP 2 K1, STK11, NOTCH1, CTNNB1, SMAD4, FBXW7* and *TP53*. The input DNA was 10 ng.

For HPV detection, real-time PCR was performed in duplicate with probes detecting HPV high-risk types 16, 18, 31, 33, 35 and 45 using tumor DNA and an established protocol used in clinical routine. Specific PCR cycling conditions are available upon request.

## Case presentation

**Fig. 1:**
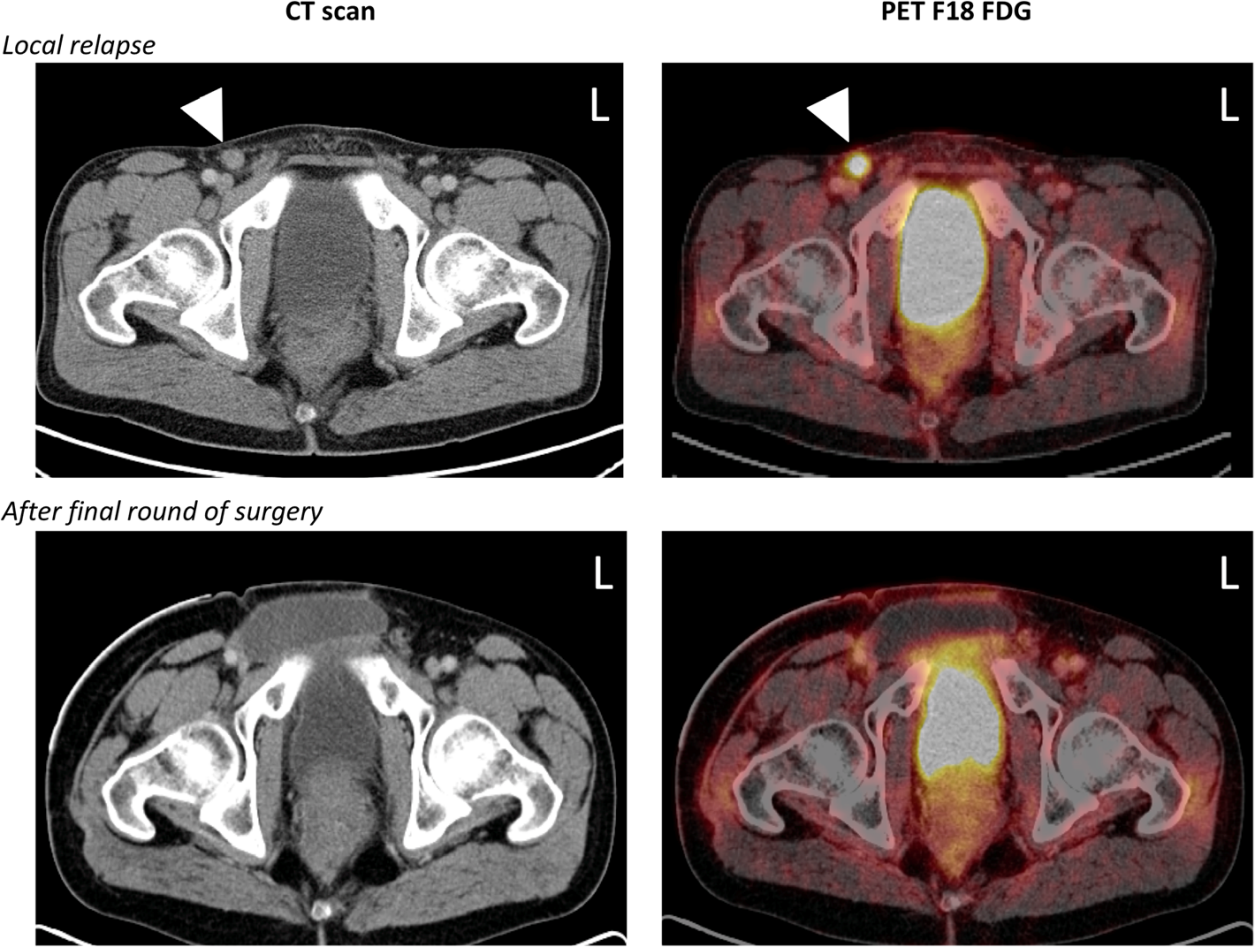
Imaging of the lower abdominal cavity and pelvic region visualizing one of the local recurrences as well as the radiological evidence of complete remission after the final round of surgery. Left columns depict conventional computerized tomography (CT) scans and right columns visualize the positron emission tomography with fluorodeoxyglucose (PET F18 FDG) findings. Top row: White arrowhead highlights one of the local recurrences, a right-sided subcutaneous inguinal lymph node metastasis. Bottom row: No remaining tumor tissue is evident after the fourth and final round of surgery. Left side is marked by the letter “L”

A 37-year-old male without any previous medical history was examined for a painful anal fissure in 2006, and a biopsy was consistent with small cell ANEC with a Ki-67 proliferation index of 80%. Laboratory with blood count, liver and kidney parameters were normal, including CEA 4.5 μg/L (reference: < 4.7). An abdominoperineal resection (APR) was performed, and the patient was subsequently treated with adjuvant cisplatin/etoposide (six rounds) as well as external radiation therapy (46.8 Gy), followed by further chemotherapy for 1 year. Two ensuing recurrences with subcutaneous lymph node metastases in the right inguinal area were noted shortly afterward, and the metastatic tissue was removed surgically in two subsequent operations 2 years after initial presentation (Fig. 1). The first metastasis measured 3.7 cm and was excised with positive margins, and the second subcutaneous relapse was 4 cm with extensive perinodal extension, almost penetrating the overlying skin. Post-operatively, the patient was administered three rounds of doxorubicin and docetaxel, with additional radiation 12.6 Gy. Soon after this, an additional subcutaneous recurrence within the abdominal wall was noted, which expanded from two to 6 cm within 2 months. Extended surgery was performed, excising a 7 cm large metastasis with negative resection margins. The resection included the spermatic cord and right testicle, but these structures were seen without tumoral engagement. This procedure was completed with reconstructive surgery using a flap technique and a skin transplant. After this, adjuvant treatment with eight rounds of irinotecan/fluorouracil and folic acid (leucovorin) were administered for 2 years. Since the last relapse 2 years after initial presentation and to our last follow-up 13 years after initial presentation, there have been no recurrences during clinical investigations and imaging performed with repeated computed tomography (CT), magnetic resonance imaging (MRI) and fluorodeoxyglucose-positron emission tomography (FDG-PET) (Fig. 1). The patient is still followed clinically by our Department of Breast and Endocrine Surgery, and a follow-up MRI of the abdomen and pelvic area is planned in the autumn of 2020. If this future radiological examination is negative for recurrences, the patient will be discharged as an outpatient. The clinical course of our patient is schematically presented in Table 1.

**Table 1:** Schematic overview of the clinical course and associated histopathological attributes

*Year*	2006	2008	2008	2008
*Surgery*	Primary surgery (abdomino-perineal resection)	Resection of first recurrence	Resection of second recurrence	Resection of third recurrence
*Diagnosis*	Primary ANEC with regional lymph node metastases	Inguinal lymph node metastasis	Inguinal lymph node metastasis	Abdominal wall metastasis
*Tumor size (cm)*	5.9	3.7	4.0	7.0
*Chromogranin A IHC*	Positive	Negative	Negative	Negative
*Synaptophysin IHC*	Positive	Positive	Positive	Positive
*Ki-67 index*	80%	90%	90%	90%
*Adjuvant chemotherapy*	Cisplatin, Etoposide	–	Doxorubicin, Docetaxel	Irinotecan, Fluorouracil
*Adjuvant radiotherapy*	46.8 Gy	–	12.6 Gy	–

*IHC* immunohistochemistry, *cm* centimeter, *ANEC* anal neuroendocrine carcinoma, *Gy* Grey, −; not administred

Histopathology of the primarily surgically resected anal lesion as well as three subsequent local recurrences was reviewed by an experienced endocrine pathologist (CCJ) to verify the original diagnoses. In this process, additional immunohistochemical and molecular analyses were ordered, and are detailed below.

**Fig. 2:**
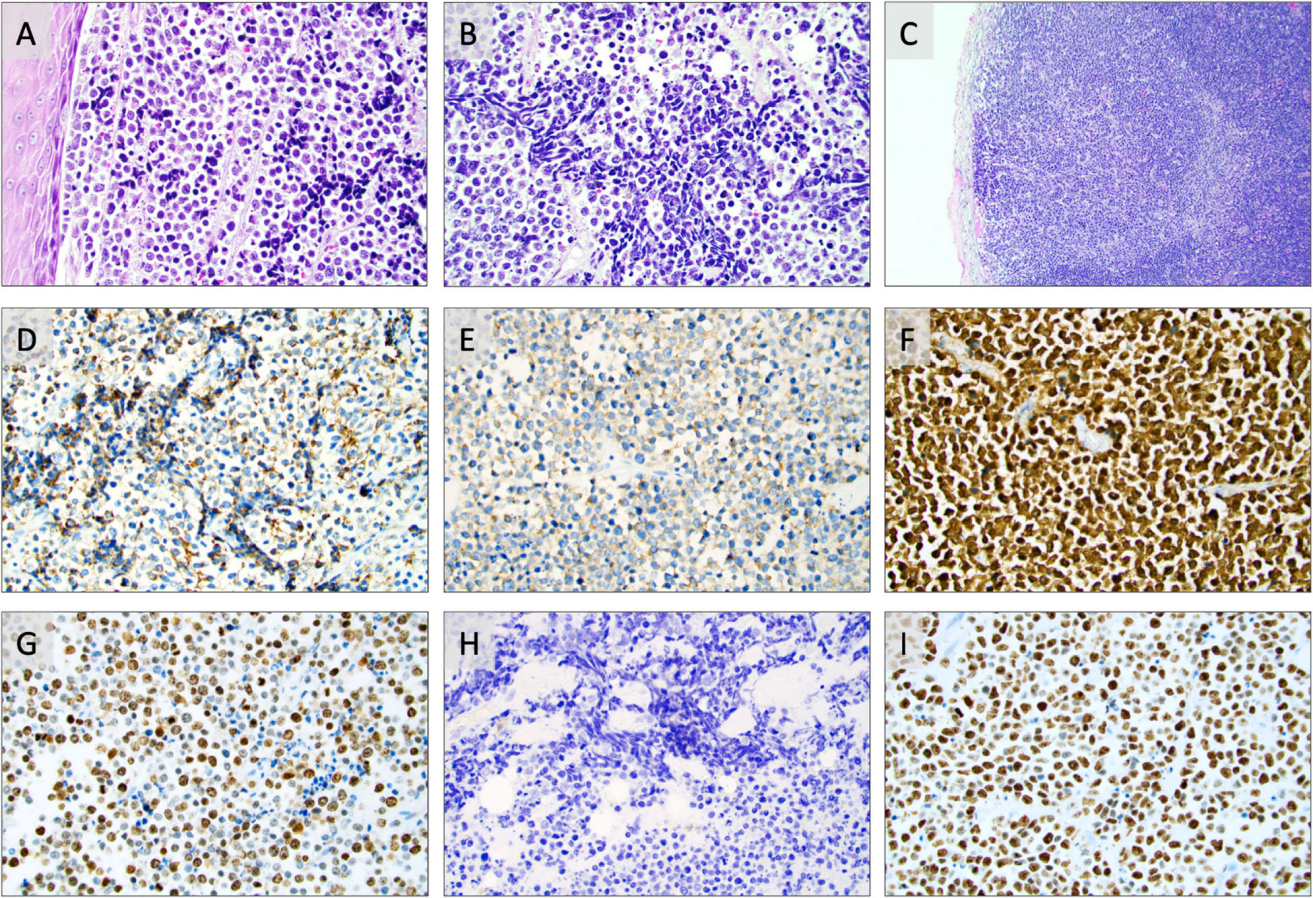
Photomicrographs of routine stained and immunohistochemical preparations of the small cell anal neuroendocrine carcinoma (ANEC). All images are magnified × 400 unless otherwise specified. **a**. Routine hematoxylin-eosin stain of the primary ANEC. The tumor is shown with solid growth and a monotonous appearance with only mild atypia. Note the squamous cell epithelium of the anal canal to the left. **b**. Routine hematoxylin-eosin stain highlighting the prominent nuclear molding, a feature of the small cell phenotype. **c**. Routine hematoxylin-eosin stain displaying a regional lymph node metastasis of the ANEC. Magnification ×100. **d-g**. Immunohistochemical analyses of the primary ANEC, displaying widespread immunoreactivity towards chromogranin A (**d**), synaptophysin (**e**), P16 (**f**) and a Ki-67 index of 80% (**g**). **h.** Regional, subcutaneous metastasis two years later displaying absent chromogranin A immunoreactivity. The same tumor was consistently positive for synaptophysin (data not shown). **j.** Same subcutaneous recurrence with a Ki-67 index of 90%

The primary anal lesion resected was found to be a 59 mm undifferentiated tumor growing in solid formations, with tumor nuclei displaying a dense chromatin and focal nuclear molding (Fig. 2a-b). Plenty of apoptotic bodies were also noted, in addition to widespread tumor necrosis and > 20 mitoses per ten high-power fields. The tumor infiltrated through the muscularis propria into the pericolic fat, but displayed negative margins. Metastases were found in 2 of 19 regional lymph nodes (Fig. 2c).

By immunohistochemistry, the tumor cells were seen positive for Chromogranin A, Synaptophysin, CD56, INSM1, OSCAR, somatostatin receptor type 2 (SSR2), P16 and focally for ISLET1 (Fig. 2d-f). Negative immunoreactivity was noted for P53, CK20, MCV-LT, CDX2, AFP, PSA, HCG, Uroplakin, GATA3, OCT3–4, PLAP, GLP-1, SOX10R and CD45. The diagnosis was consistent with a poorly differentiated small cell ANEC with a Ki-67 index of 80% (Fig. 2g). The TNM staging was pT3N1a R0.

Given the strong P16 stain, a real time PCR screening of high-risk HPV (HR-HPV) was ordered using FFPE material from the primary tumor, and HPV type 45 DNA was detected – thereby strongly suggesting an active HR-HPV infection in the excised lesion. Indeed, the combination of P16 positivity and the detection of HR-HPV DNA is a strong indicator of HPV early-gene expression [10]. Interestingly, our patient had no other known HPV associated manifestations of the genital or extra-genital regions.

When analyzing tumor DNA extracted from the primary tumor, a focused next-generation sequencing approach detected a *PIK3CA* mutation in exon 10 (c.1624G > A p.Glu542Lys;p. E542K. This specific mutation is recurrently found in carcinomas of the breast and endometrium and is therefore believed to be pathogenic. We performed an in silico analysis using PolyPhen2 (http://genetics.bwh.harvard.edu/pph2/), in which the mutation scored 0.995, indicating a high probability of this missense variant of having impact on the overall *PIK3CA* function.

Subsequent characterization of the three separate subcutaneous metastases displayed a small cell tumor with a similar histological appearance as the primary small cell ANEC diagnosed earlier. The tumor cells were now negative for Chromogranin A but positive for synaptophysin and pan-cytokeratins, and the Ki-67 index was consistently 90% in all three metastatic lesions (Fig. 2h-i). The absence of Chromogranin A expression in the subsequent relapses do not argue against an ANEC diagnosis, as reduced or absent expression of this marker is not uncommonly observed in metachronously biopsied NECs from the same individual. This reduction in sensitivity is well established and possibly reflects the underlying tumor de-differentiation process [11].

## Discussion and conclusions

We describe a patient with a highly proliferative ANEC who had surgery four times, received six different cytotoxic drugs in three combinations and has no residual disease at last follow-up 13 years after the primary diagnosis. From the first round of surgery, the tumor de-differentiated further, losing Chromogranin A expression and increasing in Ki-67 index. Intriguingly, the tumor exhibited HPV 45 DNA and strong P16 staining - suggesting the intra-tumoral presence of active HR-HPV. Moreover, sequencing analyses revealed a cancer-associated *PIK3CA* mutation. These two molecular aberrancies have never before been synchronously reported in ANECs.

ANECs are rare tumors, not least reflected in our own material. After probing our departmental pathology database using a SNOMED based search criteria with morphology codes “malignant neuroendocrine neoplasm” (M82463) and “neuroendocrine cancer” (M82493) with topography codes of “rectum” (T68000) and “anus” (T69000), we found no additional cases of ANECs over a time period of almost 30 years. However, when specifically searching through our consultation cases (second opinion reviews of tumors initially resected outside of our department), we did identify one additional patient, an 80-year-old female, with a biopsy suggesting small cell ANEC. Given the scarce availability of tissue after exhausting the FFPE block for immunohistochemical purposes, no molecular analyses had been performed. However, this tumor was also strongly P16 positive, possibly arguing for an active HPV infection also in this case (data not shown). This assumption is also in line with previous results from more comprehensive case series, in which HR-HPV DNA is recurrently detected in ANECs [5].

Complete remission of poorly differentiated NECs is indeed rare. In metastatic NECs from various sites, the median survival is 7.6 months for lung NEC, 7.5 months for gastrointestinal NEC, and 2.5 months for NEC of unknown primary [12]. However, some factors have been in favor for this patient. His age at presentation (37 years) was one of them. The median age at presentation of NECs in general is often much older [13], and sometimes concomitant diseases such as HIV or inflammatory bowel disease contributes to the poor prognosis. Our patient did not exhibit distant metastases at diagnosis, and the observed recurrences were only local with inguinal and lymph node metastases. Also, the initial surgery was performed with negative margins.

In ANEC, the molecular drivers have been poorly understood. HPV is an etiological agent of malignant diseases of the cervix, vulva, penis, anal canal, larynx as well as the head and neck region [14]. HPV is also a pathogenic factor in anal neoplasms, with up to 90% in anal SCC [15]. In individuals with HPV, viral DNA can be integrated in the genome of the host cell and this is an important route for progression of pre-neoplastic lesions to invasive carcinoma. The Rb protein is a critical negative regulator of the cell cycle that binds the transcription factor E2F and thus blocks entry into S-phase [16]. The oncoprotein E 7 from HR-HPV can cause inactivation and increase degradation of Rb, by which Rb lose its ability to halter cell cycle progression and can bring about oncogenesis in infected individuals [17].

High risk papilloma virus is associated with anal adenocarcinoma and anal squamous skin cancer [5], and HPV subtypes 16 and 18 have also been previously reported in ANEC [5, 18]. Presence of HR-HPV may also be associated to a better outcome, as in stage III anal squamous cell carcinoma in which a more favorable response to chemotherapy has been seen with a better 5-year disease free survival (DFS) [19]. If this is true also for ANECs is not known.

The dimeric enzyme kinase lipid family, phosphoinositide 3-kinases (PI3Ks), acts in regulating cellular functions as proliferation, differentiation and survival [20]. In SCC of the anal canal, *PIK3CA* mutations have been previously described [21]. Patients with genomic alterations in *PIK3CKA* have also been demonstrated in seven patients with colorectal NECs and HR-HPV type 18 [5], but if the *PIK3CKA* mutations were present in the two ANEC patients included in this study is not evident. To the best of our knowledge this is therefore the first publication where such a gene alteration has been found in anal NECs in association with HR-HPV type 45.

How to treat ANECs has been debated. Similar progression-free survival (PFS) or overall survival (OS) numbers have been found in cases treated with surgery vs. chemotherapy [8, 22]. In a publication by Brieau and colleagues, there was no difference in PFS or OS in 24 patients treated with surgery vs. chemotherapy [23]. Radiation is also a treatment possibility that should not be overlooked [24]. Debulking or resection of metastases has generally not been recommended due to the poor prognosis in metastatic disease, but remains today the only possible cure [25, 26].

Chemotherapy options as a treatment algorithm for ANECs has primarily been based on platinum-derived agents (i.e. cisplatin), which is the recommended drug of choice according to the Swedish national guidelines and the European Society for Medical Oncology (ESMO) – and was also the case for our patient [27]. This compound inhibits cell division by interference with DNA transcription and/or replication [12, 28]. In our case, cisplatin was combined with etoposide, a compound that also induces DNA damage. Even so, our patient developed metastatic recurrences despite this treatment. The second round of chemotherapy was doxorubicin (preventing DNA replication) and docetaxel (promoting tubulin assembly in microtubules and inhibits their depolymerization). By this, docetaxel acts as a mitotic poison and induces a mitotic block in proliferating cells [29]. Also, the second round of chemotherapy was largely unsuccessful. After the fourth surgical procedure, irinotecan was administered - an enzyme inhibitor blocking the enzyme topoisomerase causing double strand DNA breakage and cell death. This drug was given together with 5-fluoruracil, another enzyme inhibitor acting on thymidylate blocking formation of thymidine required for DNA synthesis [30]. During and after this treatment, following the fourth surgery, the patient has remained healthy. However, from this experience it cannot be decided if irinotecan/5-fluoruracil had an additive cytotoxic effect – but it is tempting to speculate that the combination of these drugs in association to the rare occurrence of HPV45 and/or the *PIK3CA* mutation could have influenced the disease course. Even so, we are not in a position to advocate changes to current treatment algorithms based on a single case.

Mutations in *PI3K* is frequent in most solid cancers [31], and investigations are ongoing if *PI3K/*mTOR inhibitors, *PI3K* pan-inhibitors or isoform-selective *PI3K* inhibitors could have an effect on various *PI3K* mutated tumors [32]. Of recent note, approval has been granted for the isoform-selective *PI3K* inhibitor alpelisib in the treatment of refractory breast cancer. Although speculative, such therapy may also be a potential adjuvant treatment modality for *PI3K* mutated ANECs. Ongoing studies will also clarify whether peptide receptor radionuclide therapy and/or immunotherapy with pembrolizumab or nivolumab will add to the therapeutic possibilities, not only in anal SCC but also in ANECs [33]. Moreover, vaccination against HR-HPV types 6, 11, 16, and 18 may also decrease the development of papilloma virus associated malignancies. Trials are also aiming at delivering HPV oncoprotein E6 and E7 antigens to elicit cytotoxic T-cell activation [34].

We conclude that ANEC is a rare disease. Repetitive surgery and chemotherapy may in occasional instances induce cure. The manifestation of HR-HPV type 45 and a *PIK3CA* mutation may possibly have contributed to treatment response and an overall favorable prognosis despite disseminated disease, but this has to be confirmed in future investigations.

## Data Availability

All data generated or analysed during this study are included in this published article.

## Abbreviations

APR: Abdominoperineal resection
ANEC: Anal neuroendocrine carcinoma
CT: Computerized tomography
DFS: Disease free survival
FDG-PET: Fluorodeoxyglucose-positron emission tomography
FFPE: Formalin fixated paraffin-embedded
HPV: Human papilloma virus
HR-HPV: High-risk human papilloma virus
MRI: Magnetic resonance imaging
PCR: Polymerase chain reaction
SCC: Squamous cell carcinoma

## References

[1] Lee RT, Ferreira J, Friedman K, Moss SF (2015). A rare cause of constipation: obstructing small cell neuroendocrine carcinoma of the anal canal. Int J Color Dis.

[2] Scherübl H (2009). Rectal carcinoids are on the rise: early detection by screening endoscopy. Endoscopy..

[3] WHO Classification of Tumours Editorial Board. Who classification of tumours. Digestive System Tumours. 5th ed. Lyon: IARC Press; 2019.

[4] Ohtomo R, Sekine S, Taniguchi H, Tsuda H, Moriya Y, Kushima R (2012). Anal canal neuroendocrine carcinoma associated with squamous intraepithelial neoplasia: a human papillomavirus 18-related lesion. Pathol Int.

[5] Shamir ER, Devine WP, Pekmezci M, Umetsu SE, Krings G, Federman S (2019). Identification of high-risk human papillomavirus and Rb/E2F pathway genomic alterations in mutually exclusive subsets of colorectal neuroendocrine carcinoma. Mod Pathol.

[6] Tang LH, Untch BR, Reidy DL, O’Reilly E, Dhall D, Jih L (2016). Well-differentiated neuroendocrine tumors with a morphologically apparent high-grade component: a pathway distinct from poorly differentiated neuroendocrine carcinomas. Clin Cancer Res.

[7] Bergsland EK (2013). The evolving landscape of neuroendocrine tumors. Semin Oncol.

[8] Bertani E, Ravizza D, Milione M, Massironi S, Grana CM, Zerini D (2018). Neuroendocrine neoplasms of rectum: a management update. Cancer Treat Rev.

[9] Zhang Y, Xie J, Wang J, Yang D, Jiang Z, Han G (2016). Clinicopathological and prognostic analysis of neuroendocrine carcinoma of the Colorectum. Adv Clin Exp Med.

[10] Smeets SJ, Hesselink AT, Speel E-JM, Haesevoets A, Snijders PJF, Pawlita M (2007). A novel algorithm for reliable detection of human papillomavirus in paraffin embedded head and neck cancer specimen. Int J Cancer.

[11] Bellizzi AM, Montgomery EA, Hornick JL (2020). American registry of pathology expert opinions: evaluation of poorly differentiated malignant neoplasms on limited samples - gastrointestinal mucosal biopsies. Ann Diagn Pathol.

[12] Dasari A, Mehta K, Byers LA, Sorbye H, Yao JC (2018). Comparative study of lung and extrapulmonary poorly differentiated neuroendocrine carcinomas: a SEER database analysis of 162,983 cases. Cancer..

[13] Joseph DA, Miller JW, Wu X, Chen VW, Morris CR, Goodman MT (2008). Understanding the burden of human papillomavirus-associated anal cancers in the US. Cancer..

[14] Vonsky MS, Runov AL, Gordeychuk IV, Isaguliants MG (2019). Therapeutic vaccines against human papilloma viruses: achievements and prospects. Biochemistry Mosc.

[15] Parkin DM (2006). The global health burden of infection-associated cancers in the year 2002. Int J Cancer.

[16] Giacinti C, Giordano A (2006). RB and cell cycle progression. Oncogene..

[17] Boyer SN, Wazer DE, Band V (1996). E7 protein of human papilloma virus-16 induces degradation of retinoblastoma protein through the ubiquitin-proteasome pathway. Cancer Res.

[18] Cimino-Mathews A, Sharma R, Illei PB (2012). Detection of human papillomavirus in small cell carcinomas of the anus and rectum. Am J Surg Pathol.

[19] Ravenda PS, Zampino MG, Fazio N, Barberis M, Bottiglieri L, Chiocca S (2015). Human papillomavirus in anal squamous cell carcinoma: an angel rather than a devil?. Ecancermedicalscience..

[20] Liu P, Cheng H, Roberts TM, Zhao JJ (2009). Targeting the phosphoinositide 3-kinase pathway in cancer. Nat Rev Drug Discov.

[21] Casadei Gardini A, Capelli L, Ulivi P, Giannini M, Freier E, Tamberi S (2014). KRAS, BRAF and PIK3CA status in squamous cell anal carcinoma (SCAC). PLoS One.

[22] Smith JD, Reidy DL, Goodman KA, Shia J, Nash GM (2014). A retrospective review of 126 high-grade neuroendocrine carcinomas of the colon and rectum. Ann Surg Oncol.

[23] Brieau B, Lepère C, Walter T, Lecomte T, Guimbaud R, Manfredi S (2015). Radiochemotherapy Versus Surgery in Nonmetastatic Anorectal Neuroendocrine Carcinoma: A Multicenter Study by the Association des Gastro-Entérologues Oncologues. Medicine (Baltimore).

[24] Matalon SA, Mamon HJ, Fuchs CS, Doyle LA, Tirumani SH, Ramaiya NH (2015). Anorectal Cancer: critical anatomic and staging distinctions that affect use of radiation therapy. Radiographics..

[25] Garcia-Carbonero R, Sorbye H, Baudin E, Raymond E, Wiedenmann B, Niederle B (2016). ENETS consensus guidelines for high-grade Gastroenteropancreatic neuroendocrine tumors and neuroendocrine carcinomas. Neuroendocrinology..

[26] Sorbye H, Welin S, Langer SW, Vestermark LW, Holt N, Osterlund P (2013). Predictive and prognostic factors for treatment and survival in 305 patients with advanced gastrointestinal neuroendocrine carcinoma (WHO G3): the NORDIC NEC study. Ann Oncol.

[27] Pavel M, Öberg K, Falconi M, Krenning EP, Sundin A, Perren A (2020). Gastroenteropancreatic neuroendocrine neoplasms: ESMO clinical practice guidelines for diagnosis, treatment and follow-up. Ann Oncol.

[28] Florea A-M, Büsselberg D (2011). Cisplatin as an anti-tumor drug: cellular mechanisms of activity, drug resistance and induced side effects. Cancers (Basel).

[29] Lavelle F, Bissery MC, Combeau C, Riou JF, Vrignaud P, André S (1995). Preclinical evaluation of docetaxel (Taxotere). Semin Oncol.

[30] Wigmore PM, Mustafa S, El-Beltagy M, Lyons L, Umka J, Bennett G (2010). Effects of 5-FU. Adv Exp Med Biol.

[31] Samuels Y, Wang Z, Bardelli A, Silliman N, Ptak J, Szabo S (2004). High frequency of mutations of the PIK3CA gene in human cancers. Science..

[32] Yang J, Nie J, Ma X, Wei Y, Peng Y, Wei X (2019). Targeting PI3K in cancer: mechanisms and advances in clinical trials. Mol Cancer.

[33] Morris VK, Salem ME, Nimeiri H, Iqbal S, Singh P, Ciombor K (2017). Nivolumab for previously treated unresectable metastatic anal cancer (NCI9673): a multicentre, single-arm, phase 2 study. Lancet Oncol.

[34] Yang A, Jeang J, Cheng K, Cheng T, Yang B, Wu T-C (2016). Current state in the development of candidate therapeutic HPV vaccines. Expert Rev Vaccines.

